# Risk Factors for Idiopathic Optic Neuritis Recurrence

**DOI:** 10.1371/journal.pone.0108580

**Published:** 2014-09-25

**Authors:** Yi Du, Jing-Jing Li, Yu-Jiao Zhang, Kaijun Li, Jian-Feng He

**Affiliations:** Department of Ophthalmology, The First Affiliated Hospital of Guangxi Medical University, Nanning, Guangxi, China; Institute Biomedical Research August Pi Sunyer (IDIBAPS) - Hospital Clinic of Barcelona, Spain

## Abstract

**Background:**

Approximately 30–50% of idiopathic optic neuritis (ION) patients experience one or multiple episodes of recurrence. The aim of this study was to search for risk factors for ION recurrence.

**Methods:**

Clinical data on hospitalized patients diagnosed with ION between January 2003 and January 2011 at the First Affiliated Hospital of Guangxi Medical University were retrospectively collected. Univariate and multivariate analyses were performed on factors that might cause ION recurrence. In total, 115 ION cases (32 recurrent and 83 non-recurrent cases) with complete data were analyzed. The length of the follow-up period ranged from 12 to 108 months (median: 42 months).

**Results:**

The univariate analysis showed that the recurrence rate for unilateral ION was higher than that for bilateral ION (40% vs. 12%, p = 0.001). Underlying diseases had a significant impact on recurrence (p<0.001): the recurrence rates due to neuromyelitis optica (NMO), multiple sclerosis (MS), demyelinating lesions alone of the central nervous system, and unknown causes were 89%, 70%, 41%, and 8.7%, respectively. The multivariate analysis showed that the factors causing relatively high recurrence rates included NMO (odds ratio [OR], 73.5; 95% confidence interval [CI], 7.3 to 740.9), MS (OR, 33.9; 95% CI, 5.2 to 222.2), and demyelinating lesions alone (OR, 8.9; 95% CI, 2.3 to 34.4), unilateral involvement (OR, 5.7; 95% CI, 1.5 to 21.3), relatively low initial glucocorticoid dosage (equivalent to ≤100 mg prednisone/day) (OR, 4.3; 95% CI, 1.0 to 17.9).

**Conclusion:**

Underlying diseases, laterality (unilateral or bilateral), and initial glucocorticoid dosage are important risk factors of ION recurrence. Clinical physicians are advised to treat ION patients with a sufficient dose of glucocorticoid in the initial treatment stage to reduce the recurrence risk.

## Introduction

Optic neuritis is an acute inflammatory demyelinating disease of the optic nerves [1], [2]. It can occur either independently or as a part of multiple sclerosis (MS) or neuromyelitis optica (NMO). When there are no other systemic diseases, this disease is called idiopathic optic neuritis (ION) [3]. According to literature, approximately 30–50% of ION patients have at least one recurring attack [4].

In the United States of America, the Optic Neuritis Treatment Trial (ONTT) found that unilateral ION patients receiving oral prednisone (1 mg/kg/day) faced an increased risk of new episodes of optic neuritis, while patients receiving intravenous methylprednisolone (1 g/day) did not have such an increased risk [5]. As far as we know, due to the lack of clinical studies on recurrent ION [6]–[10], we are still uncertain whether there are clinical factors, in addition to glucocorticoid dosage, that affect ION recurrence. The aim of the present study was to discover risk factors of ION recurrence.

## Methods and Patients

### 1. Ethics Statement

This retrospective study was approved by the institutional review board of the First Affiliated Hospital of Guangxi Medical University and followed the tenets of the Declaration of Helsinki. Written informed consent was not required because data were going to be analyzed anonymously. Verbal informed consent was obtained from the patients, and this was sufficient for approval by the institutional review board of the First Affiliated Hospital of Guangxi Medical University. This consent was recorded by Dr. Jing-Jing Li who explained the study procedures.

### 2. Subjects

Clinical data on hospitalized patients diagnosed with optic neuritis between January 2003 and January 2011 at the First Affiliated Hospital of Guangxi Medical University were retrospectively collected, and a follow-up study on the patients was conducted. The closing date on the follow-up was January 2012. The follow-up results were obtained through follow-up surveys over the telephone and at clinics as well as from readmission information on the patients.

In clinical practice, we diagnose optic neuritis based on the following clinical manifestations: 1) acute vision loss involving one or both eyes with or without eye pain; 2) presence of a relative afferent pupillary defect, or presence of visual evoked potential abnormalities; and 3) abnormal visual field consistent with optic neuropathy.

Patients were included if they were Chinese, more than 18 years old, and diagnosed with optic neuritis. A recurrent case was defined as follows: at least two episodes of optic neuritis attacks in the same eye with an interval between the attacks of ≥4 weeks [11]. Otherwise, the case was defined as a non-recurrent case.

Patients with the following conditions or situations were excluded from the present study: 1) ischemic, hereditary, toxic, traumatic, radiation, compressive, or infectious optic neuropathy; 2) optic neuroretinitis or optic perineuritis; 3) pre-existing MS or NMO prior to the first onset of optic neuritis; 4) other organic eye diseases and brain lesions that significantly affected visual function, such as cataracts, retinal detachment, and pituitary adenoma; and 5) lack of follow-up.

### 3. Clinical parameters

The general information on each patient, including gender, age, clinical data, imaging results, laboratory results, etc., were recorded. The follow-up was conducted to investigate the following characteristics: the effects of glucocorticoid treatment; whether optic neuritis recurred; time lapse from first onset; the number of new episodes of attack; and whether each patient converted to NMO/MS.

### 4. Visual acuity

The visual acuity of each patient was measured using a corrected decimal visual acuity. Eyes without form vision were assigned decimal equivalents as follows: counting fingers = 0.00500; hand motions = 0.00250; light perception = 0.00125 and no light perception = 0.00010 [12]. The decimal visual acuities were converted to LogMAR scores for statistical analysis. Due to the strong correlation in the visual acuity of the two eyes in patients with bilateral optic neuritis in our study (r = 0.644, p<0.001, using the Spearman correlation), the mean visual acuity (LogMAR scores) of both eyes in bilateral cases was analyzed.

### 5. Statistical analysis

SPSS 13.0 for Windows (SPSS Inc., Chicago, IL) was used for statistical analysis. The normality assumption was checked using Kolmogorov-Smirnov test. For data with a normal distribution, the Independent-Samples T Test was used for comparison; for data with a non-normal distribution, the Mann-Whitney U Test was used for comparison. The following indices were selected as enumeration data: gender, eye (unilateral or bilateral), eye pain, optic disc edema, glucocorticoid dosage, the presence or absence of demyelinating lesions of the central nervous system at the first presentation, the underlining diseases, etc. The chi-square test was used for the evaluation. After the first screening by univariate evaluation, a binary logistic regression was used to analyze the indices (p<0.2) that might potentially cause recurrence. A stepwise regression (likelihood ratio) was used to select independent variables. All the tests were two-tailed, and the significance level was set at p<0.05.

## Results

### 1. Subjects

The continuous medical records of 198 patients were collected, of which 24 records were excluded due to incomplete data (e.g., lack of visual acuity information). In total, 115 out of 174 optic neuritis cases with complete data were included in the study. Fifty-nine cases were excluded from the study because of: a lack of follow-up (40 cases); a lack of glucocorticoid treatment (2 cases); a previous diagnosis of MS prior to the first onset of optic neuritis (2 cases); Leber hereditary optic neuropathy (1 case); acquired immune deficiency syndrome (2 cases); syphilis (3 cases); macular degeneration (1 case); intracranial space-occupied lesions (2 cases); cryptococcal meningitis (1 case); and neuroretinitis (5 cases).

In the present study, no glucocorticoid-dependent patients were identified. According to the criteria proposed by Petzold and Plant [13], no patients were diagnosed with chronic relapsing inflammatory optic neuropathy.

### 2. Time data

For the 115 ION patients, the median time from the onset to confirmed diagnosis for optic neuritis was 11 days (range: 1∼30 days). For the 10 patients who eventually converted to MS, the median time from the onset of optic neuritis to MS diagnosis was 32 months (range: 0.3∼53 months). For the 9 patients who eventually converted to NMO, the median time from the onset of optic neuritis to NMO diagnosis was 14 months (range: 2∼34 months). The length of the follow-up ranged from 12 to 108 months with a median of 42 months. There were no significant differences in the duration of symptoms at admission (p = 0.470) or the length of follow-up (p = 0.074) between recurrent cases and non-recurrent cases.

### 3. Treatment and recurrence

At presentation to our hospital, 7 cases were treated with oral prednisone 40–60 mg/day, 12 cases with intravenous dexamethasone 10–15 mg/day, and 96 cases with intravenous methylprednisolone 0.5–1.0 g/day. These treatments were all followed by additional oral prednisone, which was tapered off gradually. There were 32 recurrent cases and 83 non-recurrent cases in this study. Among the 32 recurrent cases, five cases involved two new episodes of attack, two cases involved three new episodes of attack, and the remainder of the cases involved one new episode of attack. Only 3 patients received the interferon beta-1b or plasma exchange after they developed MS/NMO. No patient received disease-modifying therapies prior to the first recurrence of optic neuritis.

### 4. Univariate analysis

The age and the corrected visual acuity of the patients were subject to the normal distribution test. The age showed a normal distribution (p = 0.179), while the corrected visual acuity showed a non-normal distribution (p<0.001). The age and the corrected visual acuity were then separately subjected to univariate analyses. The age (p = 0.453) and the corrected visual acuity at admission (LogMAR scores) (p = 0.962) were unrelated to the recurrence of optic neuritis.

The results showed that eye (unilateral or bilateral) and underlying diseases were related to the recurrence of ION. Chi-square statistical analysis showed that gender, eye pain, optic disc edema, glucocorticoid dosage, and the presence or absence of demyelinating lesions at the first doctor’s visit were unrelated to ION recurrence (Table 1).

**Table 1:** Univariate analysis of risk factors for recurrence in idiopathic optic neuritis.

	Recurrence (n = 32)	Non-recurrence (n = 83)	Recurrence rate	p value
Gender				0.091*
Male (n = 35)	6	29	17%	
Female (n = 80)	26	54	33%	
Age				0.453†
Mean	36.6	38.8	-	
Standard deviation	15.0	13.6	-	
Unilateral or bilateral involvement at the first presentation				0.001*
Bilateral (n = 50)	6	44	12%	
Unilateral (n = 65)	26	39	40%	
Corrected visual acuity (logMAR scores) at the first presentation				0.962‡
Median	2.30	2.30	-	
Interquartile range	1.00–2.90	1.15–2.60	-	
Eye pain				0.398*
Yes (n = 54)	13	41	23%	
No (n = 61)	19	42	31%	
Optic disk swelling				0.098*
Yes (n = 61)	13	48	21%	
No (n = 54)	19	35	35%	
Glucocorticoid dosage in the initial treatment stage				0.128*
Equivalent to ≤100 mg prednisone/day (n = 19)	8	11	42%	
Equivalent to >100 mg prednisone/day (n = 96)	24	72	25%	
Demyelinating lesions at the first presentation				0.082*
Yes (n = 32)	14	18	44%	
No (n = 71)	16	55	23%	
Unknown (n = 12)	2	10	-	
Underlying diseases				<0.001§
Neuromyelitis optica (n = 9)	8	1	89%	
Multiple sclerosis (n = 10)	7	3	70%	
Demyelinating lesions alone (n = 27)||	11	16	41%	
Unknown cause (n = 69)	6	63	9%	

*Pearson Chi-square test.

† Independent Samples T test.

‡ Mann-Whitney U test.

§ Fisher’s Exact Test.

|| Refers only to demyelinating lesions of the central nervous system demonstrated by radiology, which do not meet the diagnostic criteria for multiple sclerosis or neuromyelitis optica.

### 5. Logistic Regression Analysis

The follow-up time and the factors (p<0.2) from Table 1 were selected as the independent variables, while the binary outcomes of “recurrence” and “non-recurrence” were used as the dependent variables. The results showed that glucocorticoid dosage, eye (unilateral or bilateral), underlying diseases were independent risk factors for recurrence (Table 2).

**Table 2:** Logistic regression analysis of risk factors for recurrence in idiopathic optic neuritis.

	Odds ratio	95% confidence interval	p value
Relatively low glucocorticoid dosage*	4.3	1.0 to 17.9	0.045
Unilateral involvement at the firstpresentation	5.7	1.5 to 21.3	0.009
Underlying diseases			
Neuromyelitis optica	73.5	7.3 to 740.9	0.000
Multiple sclerosis	33.9	5.2 to 222.2	0.000
Demyelinating lesions alone†	8.9	2.3 to 34.4	0.001
Unknown cause	1.0	-	-

*Equivalent to ≤100 mg prednisone/day.

† Refers only to demyelinating lesions of the central nervous system demonstrated by radiology, which do not meet the diagnostic criteria for multiple sclerosis or neuromyelitis optica.

## Discussion

Currently, the primary treatment for ION is the administration of glucocorticoid. The ONTT study included a 10-year follow-up on the ION patients and found that the proportion of patients with a recurrence was higher in the prednisone treatment group (44%) than in the intravenous methylprednisolone group (29%) and the placebo group (31%) [4]. Therefore, it is not recommended to treat ION patients with oral prednisone alone. However, why oral prednisone alone was associated with increased optic neuritis recurrence was not explored in the ONTT study [14]. In our study, the recurrence rate for patients who received a relatively low initial dosage (equivalent to ≤100 mg prednisone/day) was much higher than those that had a relatively high dosage (equivalent to >100 mg prednisone/day) (42% vs. 25%). In China, Zhou *et al*. treated 26 ION patients (29 eyes) with oral prednisone (initial dosage: 100–200 mg/day). They conducted a follow-up evaluation ranging from 6 months to 14 years (1 to 5 years for 18 cases) and found no recurrent cases [15].

Buttgereit *et al*. [16] proposed classifications for glucocorticoid treatment based on dosage and mechanism: (1) genomic effects: when the dosage is equivalent to ≤100 mg prednisone/day, glucocorticoid becomes effective through binding to cytosolic receptors to form complexes, which enter the nucleus and regulate target gene transcription; (2) non-genomic effects: when the dosage is equivalent to >100 mg prednisone/day, non-genomic effects also come into play, the mechanisms of which are completely different from those of the classic genomic effects. At a high doses, the cytosolic receptors are nearly completely saturated. Glucocorticoid shows non-genomic effects through reacting with cytosolic small molecular proteins and membrane/nucleus receptors, which in turn directly affect inflammatory transduction pathways.

From our calculations, we know that in the ONTT study, intravenous methylprednisolone (1 g/day) exhibited non-genomic effects, while oral prednisone alone (1 mg/kg/day) exhibited genomic effects (assuming the body weights of the patients were less than 100 kg) [5]; the mechanism of oral prednisone (100–200 mg/day) in Zhou *et al*.’s investigation involved non-genomic effects [15]. Therefore, we believe that non-genomic effects might play an important role in reducing the recurrence of ION, which warrants further investigation.

There are differences among the races and clinical characteristics of unilateral and bilateral ION patients. Unilateral ION occurs more frequently among Caucasians; the patients usually have a low proportion of optic disc edema and have a good visual acuity prognosis [5], [17]–[19]. Bilateral ION occurs predominantly among Asians and Africans, and the patients generally have a higher optic disc edema compared to unilateral ION patients; they have a relatively poor visual acuity prognosis [20]–[24]. In the present study, the recurrence rate of unilateral ION (40%) was much higher than that of bilateral ION (12%) (p = 0.001). This finding further supported that there are significant differences between unilateral and bilateral ION among Chinese patients [20].

In a previous study, ION patients were classified into three groups based on their underlining diseases: the MS group, the NMO group, and the unknown cause group [6]. In the present study, we further divided the unknown cause group and created a demyelinating lesion alone group. The patients in the demyelinating lesion alone group suffered from demyelinating lesions of the central nervous system demonstrated by radiology but did not reach the MS or NMO diagnostic criteria during the follow-up. In the ONTT study, patients with one or more demyelinating lesions on baseline brain magnetic resonance imaging (MRI) had a higher risk of developing MS (72%) than those with no demyelinating lesions (25%) [25]. Because both MS and NMO are essentially demyelinating diseases, it is predicted that the 27 ION patients with demyelinating lesions alone in the present study have an increased likelihood of eventually developing MS or NMO. In addition, the patients in the MS and NMO groups have a high recurrence rate. These findings suggest that the MS and NMO are the main cause of recurrence in ION in this study.

There is still no final conclusion on whether or not early recurrence of optic neuritis will increase the risk of developing MS or NMO. Some studies have reported that earlier and higher frequency of recurrence of optic neuritis was associated with a greater risk of developing MS [26], [27]; however, no such conclusion was obtained in one study [28]. Recurrent optic neuritis is a characteristic of NMO, but it is still unclear whether more frequent recurrence will increase the risk of developing NMO. Nevertheless, one study showed that among optic neuritis patients who were positive for serum NMO-immunoglobulin G, the hemagglutination titers of recurrent patients were higher than those of non-recurrent patients [29].

Due to limited resources, none of the patients received an NMO-immunoglobulin G test. Therefore, it is possible that some NMO patients were not diagnosed. Another limitation of this study is that there were only 19 patients in the relatively low-dosage glucocorticoid group, which might have contributed to the occurrence of edge effect (p = 0.045) when predicting recurrence; this edge effect is worth confirming in future prospective studies. However, the present study has been confirmed, based on a literature search, to have the most clinical parameters for a study on ION recurrence. Furthermore, we are the first group to elucidate whether glucocorticoid increases the risk of ION recurrence due to genomic or non-genomic effects.

## Conclusion

The present study reveals that relatively low glucocorticoid dosage (equivalent to ≤100 mg prednisone/day) and unilateral optic nerve involvement at the initial stage of the disease are factors that increase the risk of ION recurrence. The underlying diseases, including NMO, MS and demyelinating lesions alone, can dramatically increase this risk. Although there was an edge effect when using the glucocorticoid dosage for recurrence prediction in this study, the ONTT study has also demonstrated the glucocorticoid dosage would affect the recurrence rate. Therefore, it is recommended that whatever type of glucocorticoid is used to treat optic neuritis, a sufficient dosage should be given at the early stage of treatment to reduce the recurrence rate. In addition, ION patients with demyelinating lesions of the central nervous system have a high risk of recurrence. These patients may benefit from an intensified follow-up.
